# Melody Valve Implantation in the Tricuspid Position After Pediatric Heart Transplantation—A Case Report

**DOI:** 10.1016/j.jscai.2024.101354

**Published:** 2024-02-28

**Authors:** Ashish Saini, Dennis W. Kim, Kevin O. Maher, Shriprasad R. Deshpande

**Affiliations:** a Division of Pediatric Cardiology, Children’s Healthcare of Atlanta Cardiology, Emory University School of Medicine, Atlanta, Georgia; b Heart Transplant and Advanced Cardiac Therapies Program, Children's National Health Institute, Washington, DC

**Keywords:** bioprosthetic valve, case report, Melody valve, pediatric heart transplantation, tricuspid regurgitation

## Abstract

Tricuspid regurgitation (TR) is common after a heart transplant and is associated with worse clinical outcomes. The incidence ranges from 34% immediately after transplant to 20% by 10 years. Most patients can be managed medically; however, severe TR and symptomatic right heart failure warrant tricuspid valve replacement. The use of Melody transcatheter pulmonary valve in the tricuspid position is previously described. We report a unique case of posttransplant severe TR treated with surgical bioprosthetic tricuspid valve replacement who subsequently underwent successful transcatheter Melody valve placement in tricuspid position for progressive bioprosthetic valve stenosis with 11 years of follow-up.

## Introduction

The Melody transcatheter heart valve (Medtronic) is a bovine jugular venous valve sutured to a platinum-iridium stent. It is approved by the FDA and commonly employed for transcatheter pulmonary valve replacement. Transcatheter pulmonary valve replacement with a Melody valve can be performed with a high procedural success rate and low rate of complications.^1^ Long-term outcomes from the initial IDE trial have shown a 10-year freedom from valve dysfunction of 53% and freedom from any intervention of 60%.^2^ However, there are limited reports of outcomes of transcatheter Melody valve placement in the tricuspid position and none after heart transplant in children. We present a case of posttransplant progressive bioprosthetic tricuspid valve (TV) stenosis treated with transcatheter Melody valve placement with 11 years of follow-up.

## Case report

**Figure 1:**
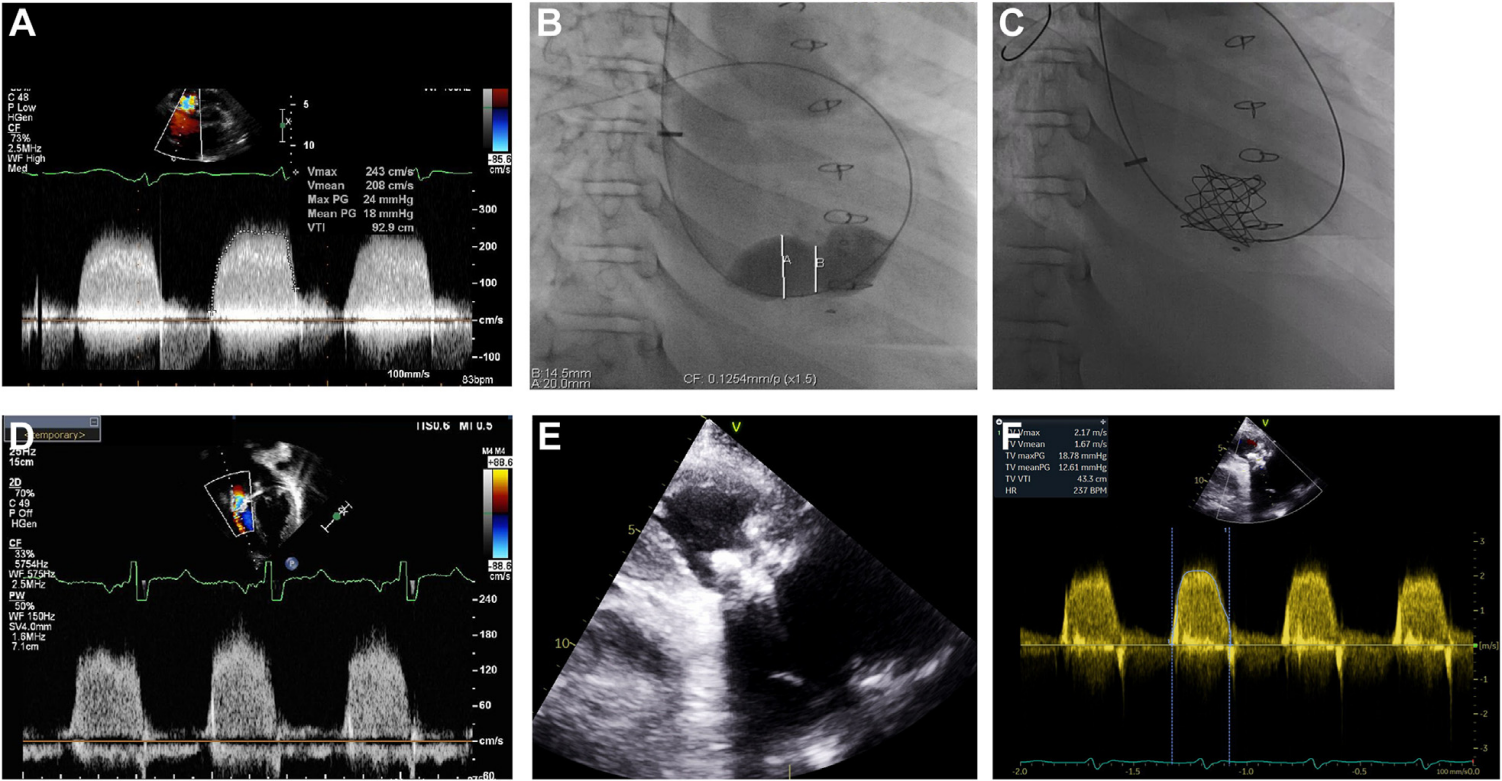
(**A**) Severe stenosis of bioprosthetic tricuspid valve. (**B**) Balloon angioplasty of bioprosthetic tricuspid valve. (**C**) Placement of Melody valve in tricuspid position. (**D**) Melody valve inflow Doppler at 5 years. (**E**) Melody valve in 2D at 11 years. (**F**) Melody valve inflow Doppler at 11 years.

At the time of writing this report, the patient was a 25-year-old man. As an infant, he was diagnosed with idiopathic dilated cardiomyopathy and underwent orthotopic heart transplantation (OHT) at 2 years of age. He developed progressive tricuspid regurgitation (TR) with a flail leaflet, probably related to the frequent need for endomyocardial biopsies. At 8 years of age, cardiac catheterization showed a central venous pressure of 20 mm Hg, mean pulmonary artery pressure of 22 mm Hg, and a pulmonary capillary wedge pressure of 16 mm Hg, in the absence of evidence of rejection. Concurrent echocardiogram showed severe TR, dilated right atrium, and right ventricle, along with depressed right ventricular (RV) function. Therefore, he underwent a surgical TV replacement with a 29-mm Mosaic bioprosthetic valve (Medtronic). The patient remained clinically well for the subsequent 5 years but developed progressive bioprosthetic TV stenosis. At 13 years of age, he underwent balloon tricuspid valvuloplasty, which resulted in a decrease in right atrial (RA) to RV end-diastolic pressure gradient from 11 to 4 mm Hg. After valvuloplasty, he developed mild to moderate bioprosthetic TV regurgitation. Progressive bioprosthetic TV stenosis and regurgitation resulted in clinical symptoms of RV failure, including the development of peripheral edema, ascites, and pleural effusion, which were refractory to medical management. Serial echocardiograms demonstrated progressive RA enlargement with a mean bioprosthetic valve inflow gradient of 18 mm Hg (Figure 1A). Based on these findings, the patient warranted TV replacement. Repeat surgical TV replacement was considered high risk due to distal graft coronary artery vasculopathy (CAV), necessitating consideration for transcatheter TV replacement. At 14 years of age, a Melody valve was positioned using a 24-mm balloon in the tricuspid position (Figure 1B, C) with no residual RA to RV end-diastolic pressure gradient. Over the subsequent 11 years of follow-up, the valve continues to maintain competency along with a valve inflow mean gradient of 12 mm Hg (Figure 1E, F). During this time, the patient has remained on low-dose aspirin. Currently, the patient is almost 24 years after transplant on maintenance immunosuppression with tacrolimus and mycophenolate and is clinically asymptomatic. Pertinent additional clinical details include stable grade 2 graft CAV, a history of electrophysiology study and ablation for atrial fibrillation, and stage 3 chronic kidney disease. Atrial fibrillation necessitated chronic anticoagulation with apixaban, starting 9 years after Melody valve implantation. The echocardiogram at the time of the last follow-up demonstrated stable but severe RA dilation, mildly reduced RV function, and normal left ventricle function. There have been no episodes of infective endocarditis.

## Discussion

TR is common after heart transplantation. TR prevalence peaks at 34% immediately after transplant, decreases to 6.4% at 3 years, and then increases to 20% by 10 years.^3^ The predominant causes are primary graft dysfunction in the early posttransplant period, graft rejection at 1 year, and longer-term complications such as CAV, and inadvertent damage due to endomyocardial biopsies.^4^ The presence of significant TR (≥moderate) is associated with worse clinical outcomes.^3^ The majority of the patients with TR are managed medically. Transplant patients with severe TR and symptomatic right heart failure may warrant TV repair or replacement. In a report of 871 patients undergoing OHT, only 17 (2%) had severe TR that required TV surgery, the majority being TV replacement.^5^ In another report of 138 patients who underwent OHT, 8 (5.8%) required TV surgery. This study explored the optimal approach for TR and reported repair for TV annular dilation and replacement for flail leaflets or chordal rupture.^6^ Our patient had severe symptomatic TR with RV failure that was addressed with surgical bioprosthetic TV replacement. Freedom from structural bioprosthetic TV valve deterioration ranges from 75% to 82.5% between 10 and 15 years.^7^ Unfortunately, our patient developed progressive bioprosthetic TV stenosis with symptoms of right heart failure. Bioprosthetic TV dysfunction has been addressed by percutaneous TV replacement using transcatheter pulmonary valves. Roberts et al^8^ reported a series of 15 patients, including 2 pediatric patients, with bioprosthetic TV dysfunction managed with percutaneous Melody valve placement with 100% procedural success. During a 4-month follow-up period, 1 patient developed third-degree heart block requiring a pacemaker and 1 developed Melody valve endocarditis.^8^ Eicken et al^9^ reported good early-term and midterm outcomes in 16 patients using Melody and SAPIEN valves for bioprosthetic TV dysfunction.^9^ The decision to use the Melody valve in our patient was guided by our experience with the valve at the time of the procedure, which was approved by FDA for pulmonary use in 2010. The Edwards SAPIEN valve had not yet been approved for transcatheter use in the pulmonary position, and our pediatric center did not have a transcatheter aortic valve replacement program. The SAPIEN valve may be more suitable for use in the tricuspid position due to its shorter length and larger diameter range.^9^ Transcatheter native TV replacement is technically challenging due to the complex anatomy of the TV apparatus and the dynamic nature of the TV annulus. A wide variety of transcatheter tricuspid-specific devices are currently being developed for use in adults.^10^ Our case is unique in utilizing percutaneous TV replacement with a Melody valve for bioprosthetic TV dysfunction in a pediatric patient post-OHT with long-term follow-up.

## Conclusion

Percutaneous valves such as the Melody valve can be effective long-term therapy for postoperative TV regurgitation or bioprosthetic TV dysfunction after heart transplantation.
